# Medical Opinion and Movement

**Published:** 1907-11-30

**Authors:** 


### Medical Opinion and Movement.

There is perhaps no disease which has participated less in the advances of medical knowledge of recent years than "rheumatoid arthritis." The discussion which took place at the Exeter meeting of the British Medical Association on the subject only served to emphasise the profound ignorance in which the profession is still groping. This ignorance is almost a reproach to medical science, for the disease has not been taken up by pathologists with the same zeal as has been displayed in other fields of research. The disease does not kill, and does not therefore perhaps make so strong an appeal to the imagination to overcome it, but a visit to any institution for incurables is sufficient to demonstrate that its crippling powers make it a real scourge to humanity. Until the pathology of the disease has passed from the stage of hypothesis into the realm of facts, an adequate method of therapy must, we fear, be despaired of. It is satisfactory, therefore, to learn that a determined effort in this direction is being made at Cambridge, and the first publication, including several papers on the subject, has just been issued. Many interesting points are brought out in regard to the ætiology of the disease; but, as in the case of cancer, we must still look to the future for the storming of the citadel that holds the vital secret of the disease.

MM. Calmette, Guerin, and Breton have for some time maintained the view that tubercular infection, even of the lungs and more distant parts of the body, takes place chiefly *via* the alimentary tract, and that in children especially the tubercle bacilli gain entrance into the body by ingestion with the food. The results of an extensive series of experiments on guinea-pigs add further confirmation to these views. An emulsion of a potato culture of the bacilli was mixed with a mucilaginous decoction of linseed, and this was introduced directly into the stomach by means of an œsophageal tube. Infection followed with great constancy, and the lesions produced were chiefly in the lungs and lymphatic glands, while the abdominal viscera were hardly ever affected. By the ingestion of bacilli killed by heat they were able to confer immunity upon guinea-pigs, so that they could tolerate with impunity at the end of two months the absorption by the digestive tract of a dose of virulent tubercle bacilli which was always fatal to the control animals. They have also experimented with bovines and have succeeded in conferring upon them a marked degree of resistance against infection by feeding with single doses of virulent bovine bacilli of a certain definite strength. The experiments were controlled by the reaction of the animals to tuberculin.

A considerable flutter has been caused by the report of the medical officer of health for the Corporation of the City of London to the effect that analyses of bottles of medicine made up according to prescriptions by chemists trading within the boundaries of the City show a high degree of inaccuracy in dispensing. This inaccuracy appears chiefly to consist in the amount of water added; correct quantities of the drugs are weighed or measured, but the water is added in sufficient quantity to fill the bottle. The assumption, however, that a 6-oz. bottle is exactly of that capacity is, according to investigations, fallacious. We understand that chemists have now taken the matter up, and guarantee that the prescription has been accurately measured into the bottle, and advise patients to use a measure glass in pouring out the doses. There is, however, a far simpler method than this. If the bottles are divided off into doses by marks on the bottle, it is of no consequence whether the total capacity is a little more or less than 6 oz. The drugs being accurately measured, if the patient takes a sixth part of the total contents each time he will obtain the prescribed dose. A question of infinitely greater importance is the quality of the drugs used. The public probably do not realise that there is as much difference in the quality of the drugs on the market as there is in tea and sugar. This is a matter which is especially worthy of consideration by the boards of hospitals and public institutions when they are considering contracts for the supply of drugs by wholesale chemists.

A problem which will doubtless receive the careful consideration of the new Medical Department of the Board of Education is the determination of the age at which infant children should be allowed to attend school, and the conditions most favourable to their healthy development. Although children are not required by law to attend school before the age of five, children under five still form about ten per cent. of all the children in the elementary schools of England and Wales. In England the age-limit for compulsory attendance is lower than in the majority of countries, where it is fixed at the age of six or seven. Dr. Arthur Newsholme dealt with the whole subject at the International Congress, and is altogether in favour of a later age-limit for school attendance. Injuries to health caused by applying infant children to near work, such as reading from books, writing and needlework, may be prevented by the institution of proper methods of infant education and supervision, but there still remain the evils of aggregation—polluted air and the risks of infection—which in his opinion outweigh the benefits derived from "social education" at these tender ages. If, however, these infant children were excluded from school, some other provision must be made for them. With the mothers out at work, and the elder children compelled to attend school, the infants cannot be kept at home beyond a certain age. In Germany, and more especially in France, the gap is filled by the formation of crèches. These would necessarily involve a considerable further outlay. It should be possible, however, to adhere to the present school arrangements, but so alter the conditions, both in regard to hygienic surroundings and methods of supervision, that infants attending shall at least not derive any physical or mental harm. Just as the health of the child is of greater importance to the community than that of the adult, so is the health of the infant worthy of even greater attention than that of the child.

